# Association between osteoporosis and hepatitis B cirrhosis: a case-control study

**DOI:** 10.4314/ahs.v20i4.13

**Published:** 2020-12

**Authors:** Yijin Zhang, Xuesong Gao, Ting Liu, Ping Gao, Hongjie Li, Nan Liu, Lili Gao, Gang Wan, Yaonan Zhang, Xuefei Duan

**Affiliations:** 1 Department of General Medicine, Beijing Ditan Hospital, Capital Medical University, Beijing, China; 2 Clinical data and sample repository, Beijing Ditan Hospital, Capital Medical University, Beijing, China; 3 Department of Medical Record, Beijing Ditan Hospital, Capital Medical University, Beijing, China; 4 Department of Orthopedics, Beijing Hospital, National Center of Gerontology, Beijing, China

**Keywords:** Liver cirrhosis, bone density, osteoporosis, osteopenia, hepatitis B, chronic

## Abstract

**Background and aims:**

Hepatitis B virus (HBV)-related cirrhosis is associated with decreased bone mineral density (BMD); however, the mechanism is yet unknown. To assess the incidence of osteoporosis in patients with HBV-associated cirrhosis and relevant mechanisms.

**Methods:**

A total of 80 hospitalized patients with HBV-associated cirrhosis and 80 healthy controls were enrolled. The levels of serum osteocalcin, total procollagen type 1 amino-terminal propeptide, β-C-terminal telopeptide of type I collagen (β-CTX), and 25-hydroxy vitamin D3 (25(OH)D3) was evaluated in the cirrhosis group.

**Results:**

The BMDs of the lumbar spine (P<0.001) and hip joints (P=0.015) in the cirrhosis group were significantly lower than those in the controls. The incidence of osteoporosis in the cirrhosis group was significantly higher than that in the control group (P<0.001). Compared to the patients of the Child-Pugh grade A and B, the BMD of lumbar spine and 25(OH)D3 was significantly decreased in patients of grade C, while β-CTX was elevated. Patients in the cirrhosis group faced a higher risk of osteoporosis as compared to the controls(P<0.001).

**Conclusions:**

Enhanced bone resorption accounted for increased risk of osteoporosis in severe cirrhosis. Thus, HBV-associated cirrhosis was a risk factor for osteoporosis.

## Introduction

Approximately 248 million individuals are affected by chronic hepatitis B (CHB), globally. The number of patients with HBV-associated cirrhosis, hepatic decompensation, and deaths was 371,100 in 2015 1. Hepatic osteodystrophy is a non-liver complication of chronic liver disease, especially in patients with cirrhosis. Osteoporosis is the most common form of hepatic osteodystrophy and is associated with the increased risk of fragility fractures that lead to social and economic burdens^2^. Vertebral fractures are the most common types in patients with chronic liver disease^3^.

Recent studies mainly focused on the association between primary biliary cirrhosis or alcoholic cirrhosis and osteoporosis^4^–^6^. The pathogenesis of CHB patients with cirrhosis complicated with osteoporosis has not yet been well studied.

Thus, the present study aimed to evaluate the correlation between decreased bone mineral density (BMD) and bone markers to explore the related pathogenesis in CHB patients with cirrhosis.

## Subjects and methods

### Study design

In this case-control study, 80 CHB patients with cirrhosis hospitalized in the Department of General Medicine of Beijing Ditan Hospital of Capital Medical University between March 2015 and 2017 were selected as the subjects. Te inclusion criteria included patients with hepatitis B cirrhosis who were diagnosed by laboratory examination, histology, or imaging according to the guidelines of the American Association for the Study of Liver Diseases^7^. Also, the medical history of the patients including smoking history, age at menopause, history of drug abuse, family history of osteoporosis, eating habits, comorbidities, and history of antiviral therapy with HBV was collected. In addition, 80 healthy individuals were enrolled as controls from the Beijing Hospital Health Examination Center. The exclusion criteria for both groups were as follows: diseases affecting bone metabolism (for instance, parathyroid diseases, thyroid diseases, inherited disorders of connective tissue, sarcoidosis, or inflammatory bowel disease), patients withdual-energy X-ray absorptiometry(DXA) contraindications, patients taking estrogens or proton pump inhibitors, any medications that could affect the bone mass (for example, calcium, vitamin D supplements, corticosteroids), immunosuppressant therapy without organ transplant, history of malignant tumor, severe heart disease, chronic renal insufficiency, human immunodeficiency virus (HIV) co-infection, hepatitis C virus co-infection, autoimmune liver disease, primary biliary cirrhosis, and alcoholic liver disease.

This study was approved by the Institutional Review Board of the Beijing Ditan Hospital, Capital Medical University. Written informed consent was obtained from all participants (2015-041-01).

### BMD measurements and laboratory examination

BMD (g/cm2) of the lumbar spine and the proximal hip (femoral neck, trochanter, and total hip) were measured by dual-energy X-ray absorptiometry (DXA) (QDR-4500A; Hologic, Waltham, MA, USA). Osteoporosis was defined as a T score below –2.5 standard deviations (SD), while osteopenia was defined as a T score between –1 and –2.5 SD 8.

The levels of serum osteocalcin (OC), β-C-terminal telopeptides of type-I collagen (β-CTX), procollagen type 1 amino-terminal propeptide (P1NP), and 25-hydroxyvitamin D3 (25(OH)D3) were measured using electrochemiluminescence immunoassay (Roche Diagnostics GmbH, Mannheim, Germany). β-CTX is a degradation product of type 1 collagen and released into the circulation during bone resorption^9^. On the other hand, procollagen type 1 amino-terminal propeptide (P1NP) is a bone formation marker that is released into the circulation during bone formation^9^. Osteocalcin is a polypeptide secreted primarily by osteoblasts into the bone matrix, and hence, is considered to be a marker of bone formation(10). 25(OH)D3 reflects the nutritional level of vitamin D.

Child-Pugh scores were used for the clinical staging of cirrhosis: A (5–6), B (7–9), and C (10–15), including five indicators: hepatic encephalopathy (phase), ascites, total bilirubin (µ mol/L), albumin (g/L), and prothrombin time extension (s). The total bilirubin and albumin levels were tested using a Hitachi 7600 fully automatic biochemical analyzer (Wako Pure Chemical Industries Ltd, Tokyo, Japan). The prothrombin time was tested using an automatic blood coagulation instrument (CA6000, Sysmex Corporation, Japan).

## Statistical analysis

Quantitative data were expressed as mean±SD. Qualitative data were reported as proportion (%). The chi-square test was used for categorical variables. Student's t-test was utilized to compare data between two groups, and the Kruskal-Wallis test was used for comparisons across three groups with respect to continuous variables. P<0.05 was considered statistically significant. Logistic regression analysis was used for the risk factors of osteoporosisanalyzed by the stepwise regression method, and the variables were selected using the backward method. All statistical tests were carried out by SPSS version 17 (SPSS Inc., Chicago, IL, USA).

## Results

The clinical characteristics of the HBV cirrhotic patients and controls are summarized in Table 1. The cohort consisted of 44 (55.0%) males and 36 (45.0%) females in the HBV cirrhotic group and 40 (50%) males and 40 (50%) females in the control group. The mean age of the control group (54.7±10.8, range 31–75 years) did not differ significantly from that of the HBV cirrhotic patients group (51.7±9.6; range 31–75 years) (P=0.069). The mean BMI of the control group (24.54±3.14, range 18.3–34.2kg/m2) was not significantly different from that of the HBV cirrhotic patient group (23.88±4.20, range 16.7–34.1 kg/m2) (P=0.224). 25% (20/80) of the patients with hepatitis B cirrhosis and 20% (16/80) in the healthy control group smoked. In the HBV cirrhosis group, 75% (27/36) and 62.5% (25/40) women in the control group had menopause.

**Table 1:** Characteristics of patients

Variables	HBV cirrhosis	Controls	P-value
	(n=80)	(n=80)	
Age (years, mean±SD)	51.7±9.6	54.7±10.8	0.069
Male, n (%)	44 (55.00)	40 (50.00)	0.527
BMI (kg/m^2^, mean±SD)	23.88±4.20	24.54±3.14	0.224
Smoke, n (%)	20 (25.00)	16 (20.00)	0.449
Menopause, n (%)	27 (75.00)	25 (62.5)	0.242

Herein, we found that 70% (56/80) of the HBV cirrhotic patients had decreased BMD as compared to 35% (28/80) of the control group. Compared to the control group, the HBV cirrhotic patients had significantly higher rates of osteoporosis (27.5% vs. 7.5%, P<0.001). In the HBV cirrhotic group, 5 patients experienced fragility fractures; one of these patients suffered right wrist and left ankle fractures, respectively, while no fractures occurred in the control group. The BMD values of the lumbar spine, femoral neck, trochanter, and total hip in the HBV cirrhotic patients and controls are shown in Table 2. The BMD values in all scanned regions were significantly lower in the HBV cirrhotic patients as compared to the controls. Furthermore, the BMD was lower in the patients in the C grade subgroup as compared to the A and B grade subgroups, and the lumbar spine BMD was significantly lower in the C grade subgroup than that in the A and B grade subgroups. The femoral neck, trochanter, and total hip BMD were also lower in the Child-Pugh C subgroup, albeit not significantly (Table 3).

**Table 2:** Bone mineral density in the HBV group and the control group

Site	BMD(g/cm^2^) HBV cirrhosis (n=80)	BMD(g/cm^2^) Controls (n=80)	P-value
LS	0.90±0.15	1.00±0.14	<0.001
FN	0.72±0.12	0.76±0.11	0.029
WT	0.65±0.10	0.69±0.09	0.006
Tro	1.08±0.16	1.13±0.14	0.040
TH	0.89±0.13	0.93±0.11	0.015

LS: lumbar spine; FN: femoral neck; Tro: trochanter; TH: total hip

**Table 3:** Association between BMD and Child-Pugh grade of HBV cirrhotic patients

Site (g/cm	BMD(g/cm^2^) Child-Pugh A (n=57)	BMD(g/cm^2^) Child-Pugh B (n=16)	BMD(g/cm^2^) Child-Pugh C (n=7)	P-value
LS	0.90±0.14	0.93±0.14	0.77±0.19	0.039
FN	0.73±0.12	0.72±0.12	0.68±0.11	0.567
WT	0.66±0.11	0.66±0.12	0.59±0.07	0.296
Tro	1.08±0.16	1.08±0.19	1.04±0.17	0.763
TH	0.89±0.13	0.90±0.15	0.81±0.13	0.269

LS: lumbar spine; FN: femoral neck; Tro: trochanter; TH: total hip

Serum β-CTX was significantly higher in the Child-Pugh C grade subgroup than in the A and B grade subgroups, and 25(OH)D3 was significantly lower in the Child-Pugh C grade subgroup than in the A and B grade subgroups. The levels of P1NP and OC were higher in the Child-Pugh C grade subgroup, but the difference was not statistically significant (Table 4).

**Table 4:** Association between bone markers and Child-Pugh scores in HBV cirrhotic patients

Variable (ng/mL)	Child-Pugh A (n=57)	Child-Pugh B (n=16)	Child-Pugh C (n=7)	P-value
P1NP	60.53±42.01	47.96±23.31	76.28±17.98	0.236
OC	18.19±9.69	16.64±10.76	18.59±9.15	0.841
β-CTX	0.47±0.28	0.57±0.40	1.01±0.59	0.001
25(OH)D25(OH)D3	21.00±10.42	15.73±7.35	12.38±8.62	0.029

P1NP: procollagen type 1 amino-terminal propeptide; OC: serum osteocalcin; β-CTX: β-C-terminal telopeptides of type-I collagen; 25(OH)D3: 25-Hydroxyvitamin D3

Multivariate logistic regression analysis was performed in the presence of osteoporosis as the dependent variable and gender, age, BMI, smoking, and hepatitis B as independent variables. The results showed that age and hepatitis B were risk factors for osteoporosis. Interestingly, the risk of osteoporosis in patients with chronic hepatitis B was higher than that in healthy controls (Table 5).

**Table 5:** Risk factors of osteoporosis in patients with chronic hepatitis B

Effect	P-value	OR	95% CI for OR	

			Lower	Upper
Gender	0.004	0.191	0.061	0.599
Age	0.003	1.056	1.019	1.094
BMI	0.025	0.895	0.813	0.986
Smoking	0.133	1.815	0.834	3.950
HBV	0.000	5.299	2.566	10.943

## Discussion

The current study demonstrated that the prevalence of osteoporosis (27.5%) was higher in CHB patients with cirrhosis as compared to the controls. The progression of cirrhosis from A to C grades of the Child-Pugh classification was associated with a decreased BMD, which was consistent with the literature^11^. The lumbar spine BMD was significantly lower in the Child-Pugh C grade subgroup than that in the A and B grade subgroups in the cirrhotic patients. In addition, β-CTX was elevated with the progression of cirrhosis.

Recently, a large-scale population-based cohort study from Taiwan demonstrated that chronic HBV infection increases the risk of osteoporosis^12^. Interestingly, the risk remained higher in the cohort, even after adjusting for age, sex, and complications of cirrhosis, chronic renal insufficiency, thyroid diseases, diabetes, and hypertension^12^. In the current study, 5/80 patients had experienced fragility fractures in the cirrhotic group, while no patient had a fracture in the control group. The association between HBV infection and osteoporotic fractures was statistically significant. However, Chen et al. showed that the incidence of osteoporotic fractures was not statistically higher in patients with HBV as compared to those without HBV infection^12^. Intriguingly, Byrne et al. found that chronic HBV infection increased the risk of hip fracture in the patients of all races except for Asians^13^. The differences between these studies highlighted why the patients enrolled in our study were all cirrhotic patients. HBV infection was a less important risk factor for osteoporosis as compared to cirrhosis. Therefore, a large-scale study is essential to further explore the correlation between HBV-related cirrhosis and osteoporotic fractures.

In the present study, β-CTX was significantly higher in cirrhotic patients of Child-Pugh grade C. We hypothesized that severe cirrhotic patients exhibit accelerated bone resorption. The lumbar spine BMD decreased with the progression of cirrhosis. The mechanism of association between the decreased BMD and severity of cirrhosis has not yet been fully elucidated. The production of inflammatory cytokines, such as interleukin-6 (IL-6), tumor necrosis factor-alpha (TNF-α), and IL-1 increases the receptor activator of the nuclear factor kappa-B ligand, which further promotesosteoclastogenesis and bone resorption, resulting in a decrease in BMD^14^–^17^. Hepatic decompensation or cirrhosis also prevents bone formation by impairing the production of 25(OH)D3 and insulin-like growth factor-1 18, 19. Moreover, hypogonadism with decreased levels of estrogen and testosterone accelerates the bone loss, primarily due to the increased osteoclast activity, which is often observed in the decompensated livers^20^. Furthermore, a decompensated liver impairs the collagen-binding of the bone matrix and inhibits osteoblast function^21^. Finally, increased bilirubin inhibits osteoblast proliferation^22^–^24^. Also, we provided data on the changes in 25(OH)D3 and β-CTX and their clinical significance in CHB patients with cirrhosis. A remarkably reduced level of 25(OH)D3 was observed in cases of severe cirrhosis. Similar results have been reported in a previous study of cirrhotic patients^25^. The possible underlying mechanisms include decreased hepatic hydroxylation of vitamin D, intestinal malabsorption, lessened sunlight exposure, and reduced dietary intake. Vitamin D deficiency decreases calcium resorption and activates bone resorption to maintain the level of calcium in the blood, resulting in bone loss^26^. In addition, impaired hydroxylation of vitamin D3 to 25-OH-D3 in the liver promotes bone loss and decreased bone formation^27^. The increased osteoclast function and bone resorption partially explained the high rates of low BMD in cirrhosis. Regression analysis showed that age and hepatitis B were risk factors for osteoporosis. Therefore, the diagnosis and treatment of osteoporosis in CHB patients should be investigated to prevent osteoporosis and retain the quality of life of the patients.

The major strength of the current study was that bone turnover markers were evaluated as potential mechanisms of bone disease in HBV-related cirrhotic patients. A major limitation of this study was the cross-sectional design, although the changes in BMD were estimated longitudinally. Also, whether the results could be extrapolated to all the cirrhotic patients owing to the small sample size of the Child-Pugh grade C cirrhotic patients was unclear. Furthermore, the controls did not present results for the bone turnover markers.

## Conclusion

Osteoporosis was highly prevalent among CHB patients with cirrhosis. Child-Pugh grade C cirrhotic patients showed enhanced osteoclastic activity, indicated by increased β-CTX. Enhanced bone resorption accounted for the increased risk of osteoporosis in patients with severe cirrhosis. Thus, osteoporosis in HBV-related cirrhotic patients should be under intensive focus. CHB patients diagnosed first time with cirrhosis should undergo DXA examination for the early diagnosis of the onset of osteoporosis. Therefore, patients with cirrhosis are recommended to undergo BMD assessments annually. After osteoporosis is diagnosed in cirrhotic patients, drug therapy that inhibit the activity of osteoclasts must be prescribed to reduce the risk of fractures.
